# Metallo supramolecular cylinders inhibit HIV-1 TAR-TAT complex formation and viral replication *in cellulo*

**DOI:** 10.1038/s41598-018-31513-3

**Published:** 2018-09-06

**Authors:** Lucia Cardo, Isabel Nawroth, Peter J. Cail, Jane A. McKeating, Michael J. Hannon

**Affiliations:** 1 School of Chemistry, University of Birmingham, Edgbaston, Birmingham, B15 2TT UK; 2 Institute of Immunology and Immunotherapy Centre for Human Virology, University of Birmingham, Edgbaston, Birmingham, B15 2TT UK; 3 Nuffield Department of Medicine, Oxford University, Oxford, OX3 7BN UK

## Abstract

Shape-selective recognition of nucleic acid structures by supramolecular drugs offers the potential to treat disease. The Trans Activation Response (TAR) region is a region of high secondary structure within the human immunodeficiency virus-1 (HIV-1) RNA that complexes with the virus-encoded Transactivator protein (TAT) and regulates viral transcription. Herein, we explore different metallo-supramolecular triple stranded helicates (cylinders) that target the TAR bulge motif and inhibit the formation of TAR-TAT complexes and HIV infection. Cylinders that incorporate Ni(II) and Ru(II) showed the most potent anti-viral activity with limited evidence of cellular cytotoxicity. These metallo-supramolecular compounds provide an exciting avenue for developing a new class of anti-viral agents.

## Introduction

Ribonucleic acid (RNA) is involved in a wide range of biological events, mediating the processing of genetic information from DNA to proteins, as structural components of many ribonucleoproteins and as non-coding elements with a variety of gene regulatory functions^1,2^. Microrganisms, including diverse fungi, bacterial and viral species encode RNA species with distinctive 3-dimensional structures that play a role in their replicative life cycles and represent attractive targets for anti-microbial research^3^. However, RNA recognition by small molecule drugs is poorly developed compared to the large number of synthetic agents that target DNA motifs and proteins. The diversity and dynamic nature of RNA structures, reflecting its multiple functions, is a major challenge in designing RNA binding molecules, yet offers the potential for shape recognition of structural features of selected RNAs, providing a strategy in drug development and diagnostics^3–5^.

**Figure 1:**
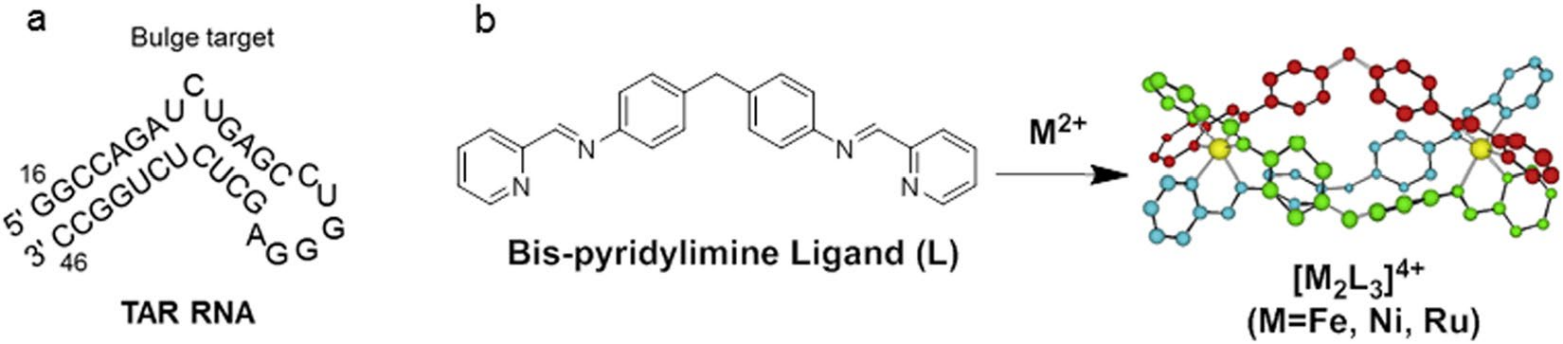
HIV TAR RNA and cylinders. (**a**) The HIV TAR RNA sequence and (**b**) di-nuclear triple stranded helicate (cylinder) obtained upon reaction of bis-pyridylimine ligand L with octahedral metals (i.e. Fe^2+^, Ni^2+^ and Ru^2+^).

The retrovirus human immunodeficiency virus type 1 (HIV-1) encodes a Trans Activation Response (TAR) RNA element that comprises a conserved 59-nucleotide stem-loop structure located within the 5′ end of the transcribed viral mRNA (Fig. 1a) in the long terminal repeat (LTR). The three nucleotide bulge region of TAR interacts with the viral encoded transactivator protein (TAT) and forms a complex, together with other cellular cofactors, that regulates viral transcription. Hence, the TAT-TAR complex provides an interesting target for anti-retroviral therapy (ART)^6,7^. Current therapies for treating HIV infection target viral encoded enzymes that play an essential role in the replicative life cycle and include reverse transcriptase, protease and integrase^8^. Given the error prone nature of the reverse transcriptase coupled with a high replicative capacity, HIV exists as a quasispecies of viral variants that can develop resistance to anti-viral therapies. Efficient ART requires drug combinations targeting multiple enzymes in the viral life cycle. The highly conserved HIV TAR motif provides an attractive target for the design of small molecules that inhibit TAR-TAT complex formation; however, to date there has been a paucity of agents targeting this step in the viral life cycle.

The diverse 3D shapes of transition metal-based compounds, and their cationic charge make them ideal compounds to target specific nucleic acid structural motifs^9^, although most of the examples reported to date focus on binding DNA; by comparison, the field of RNA recognition by metal complexes is unexplored^10^. There are only a few examples of metal-based-systems that target HIV TAR RNA^11–16^ or are conjugated to TAR binding organic compounds^17^. We previously reported a class of metallo-helicates^18–23^, also known as ‘cylinders’, that can bind DNA and RNA 3-way junctions^19,22^ and bulge structures^23^. We also reported these cylinders to have cytostatic and cytotoxic activity against mammalian cancer cell lines^20,24^, and bactericidal properties^25^. In particular, the iron cylinder (FeCy) comprising three strands of bis-pyridylimine ligands that coordinate two iron(II) atoms (Fig. 1b), was reported to bind the bulge site of TAR RNA *in vitro* and to limit TAR-ADP-1 complex formation^26^, where ADP-1 is a synthetic peptide representing the region of TAT that binds TAR^27,28^. However, this biophysical observation requires validation to show anti-viral activity. The reported toxicity of iron(II) cylinders for mammalian cancer cell lines^24^ and ability of iron(II) to promote HIV infection^29,30^, limits the potential application of this compound.

In this paper, we address these challenges and demonstrate that alternative metal containing supramolecular cylinders bind TAR RNA and reduce ADP-1 interaction(s) and importantly inhibit HIV replication in cell based systems with no significant toxicity.

## Results and Discussion

We employed nickel(II) and ruthenium(II) triple stranded helicates^20,31^ (NiCy and RuCy, Fig. 1b) that are both iso-structural and iso-tetracationic with FeCy, but have increased kinetic stability. Metal (II) triple stranded cylinders bind specific nucleic acid motifs through electrostatic interactions and π−stacking between bis-pyridylimine ligands and nucleobases^18,19,22^, and we predict similar binding affinities to HIV TAR RNA.

**Figure 2:**
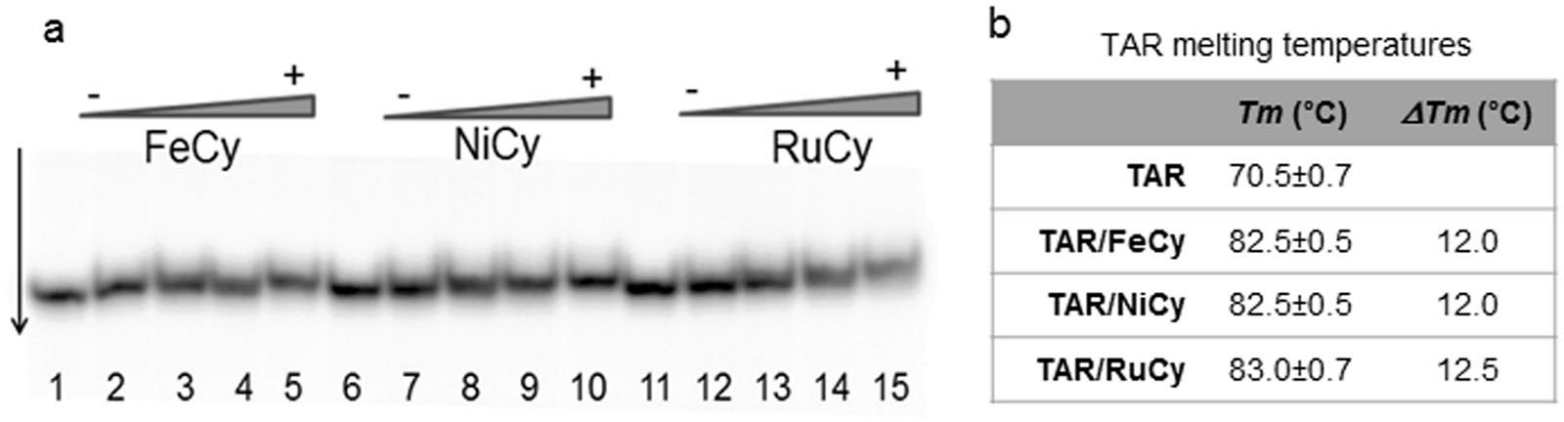
Cylinders bind HIV TAR. (**a**) Autoradiograph of ^32^P labelled HIV TAR RNA (2 µM) alone (lanes 1, 6 and 11) or following incubation with increasing concentrations (1, 2, 3 and 4 µM) of FeCy (lanes 2–5), NiCy (lanes 7–10) or RuCy (lanes 12–15). (**b**) Melting Temperatures (***Tm***) and corresponding melting temperature increases (***ΔTm***) of TAR RNA (3 μM) alone and in the presence of the three cylinders (TAR:Cylinder at 1:1 ratio) calculated from UV melting analyses (details of RNA melting curves are in Figure S1).

**Figure 3:**
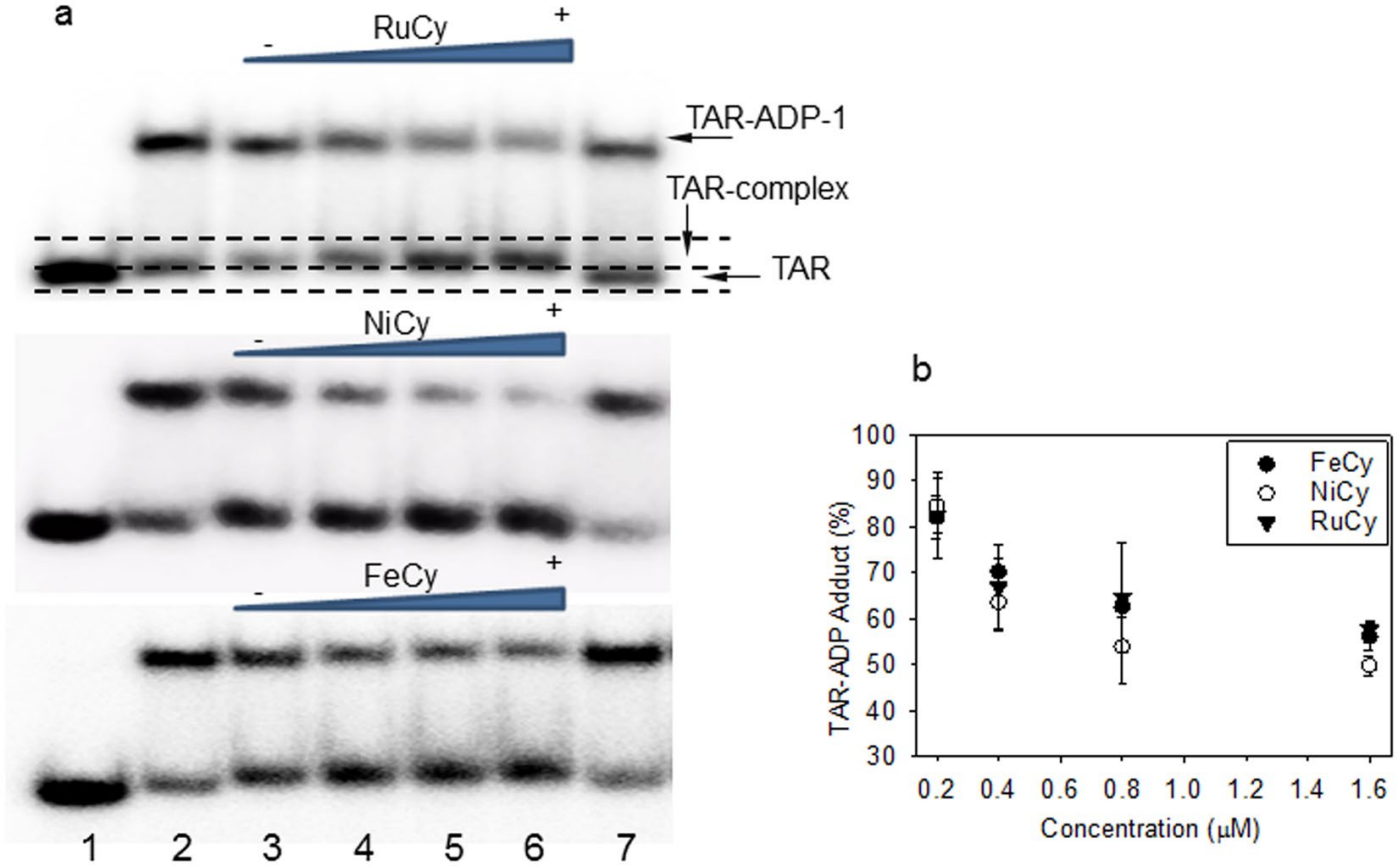
Cylinders inhibit HIV TAR-ADP-1 complex formation. (**a**) lane 1: ^32^P labelled HIV TAR RNA (0.1 µM); Lanes 2 and 7: 0.1 µM of TAR pre-incubated with 0.3 µM of ADP-1 (the top band corresponds to TAR-ADP-1 adduct); lanes 3–6, TAR and ADP-1 incubated with increasing concentration of FeCy, NiCy and RuCy (0.2, 0.4, 0.8, and 1.6 µM); TAR-ADP-1 adduct is replaced by TAR-Cy adducts (middle and bottom autoradiograms were cropped from the full-length versions in S4). (**b**) Densitometric analysis of TAR-ADP-1 adducts compared to untreated samples (data are the average of three independent gels ± s.d.); all cylinders similarly inhibit TAR-ADP-1 (P values > 0.05, calculated by one way Anova analysis between three y values at each concentration).

Indeed, in electrophoretic mobility shift assays, FeCy, NiCy and RuCy cylinders show comparable binding activity for the HIV TAR RNA sequence. Incubating TAR RNA with an increasing concentration of each cylinder for 30 minutes increased TAR-cylinder adducts (Fig. 2a). The shift in band mobility is subtle, as reported with other 3 way junctions and bulges, and is consistent with the cylinder locating within the centre of the bulge^23,26^. We further confirmed equivalent binding properties of the three cylinders toward TAR RNA, by performing thermal stability and fluorescent intercalator displacement (FID) assays. Analysis of UV melting curves (Figs 2b and S1) shows that each cylinder equally stabilises TAR RNA, by inducing an increase of melting temperature (***ΔTm***) of 12 °C. In FID assays, all three cylinders displace ethidium bromide from TAR RNA, with analogous similar displacement curves and DC_50_ values (Figure S2). Crucially, all three cylinders inhibit TAR-ADP-1 complexes in a concentration-dependent manner (Fig. 3 and Figure S3) and with comparable efficiencies, showing a ~50% reduction in complexes at a cylinder/TAR ratio of 16:1 ratio (Fig. 3b) and confirming cylinders bind at the bulge region.

**Figure 4:**
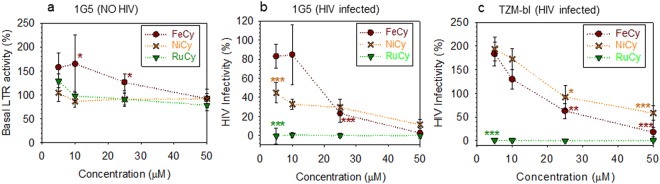
Cylinders inhibit HIV replication. **(a)** Uninfected 1G5 cells were treated with an increasing concentration of cylinders for 48 h and basal LTR activity measured. 1G5 **(b)** or TZM-bl cells **(c)** were treated with an increasing concentration of cylinders for 4 h before infecting with HIV-1 NL4.3 for 48 h. Data is presented as percentage relative to control (cells in absence of cylinders, corresponding to 100% in each graph) and represents the mean ± s.d. of six biological samples. Each y value was compared with the corresponding control by one way ANOVA analysis followed by Bonferroni post hoc test and P values < 0.05 indicate where their difference is statistically relevant. (*P < 0.05, **P < 0.01, ***P < 0.001).

Having demonstrated that all cylinders equally bind TAR and inhibit ADP-1 binding *in vitro*, we investigated their ability to inhibit HIV replication by using two independent reporter cell lines, 1G5 Jurkat T cells and TZM-bl HeLa cells, both expressing the HIV LTR gene coupled to a firefly luciferase gene. Incubation of these cells with the active virus promotes expression of the LTR followed by the luciferase gene, generating a chemiluminescent signal that is proportional to the viral replicative burden^32,33^. We initially assessed the effect of cylinders on basal LTR activity in 1G5 cells in absence of the virus (uninfected cells, Fig. 4a). Ni and Ru cylinders had no detectable effect on basal promoter activity whereas FeCy induced a modest 1.5–2 fold increase in luciferase at the lowest concentration (5–10 µM) tested. As a further control, we demonstrated that NiCy and RuCy absorbance (in the visible) had no quenching effect on luciferin emission and they were not interfering with this luminescence based assay (Figure S5).

Next, we evaluated the ability of cylinders to prevent HIV-1 infection: 1G5 and TZM-bl cells (Fig. 4b,c respectively) were treated with an increasing dose of cylinders for 4 h prior to infecting with HIV-1 NL4.3 for 48 h. Both NiCy and RuCy inhibited HIV replication with RuCy showing the more potent anti-viral activity in both cell lines. In contrast, FeCy had a minimal effect on HIV replication and only at highest doses. In summary these results show that Ni and Ru containing cylinders inhibit HIV replication in a TAT-dependent manner, with RuCy showing >95% efficiency already at 5 μM (incubation concentration).

**Figure 5:**
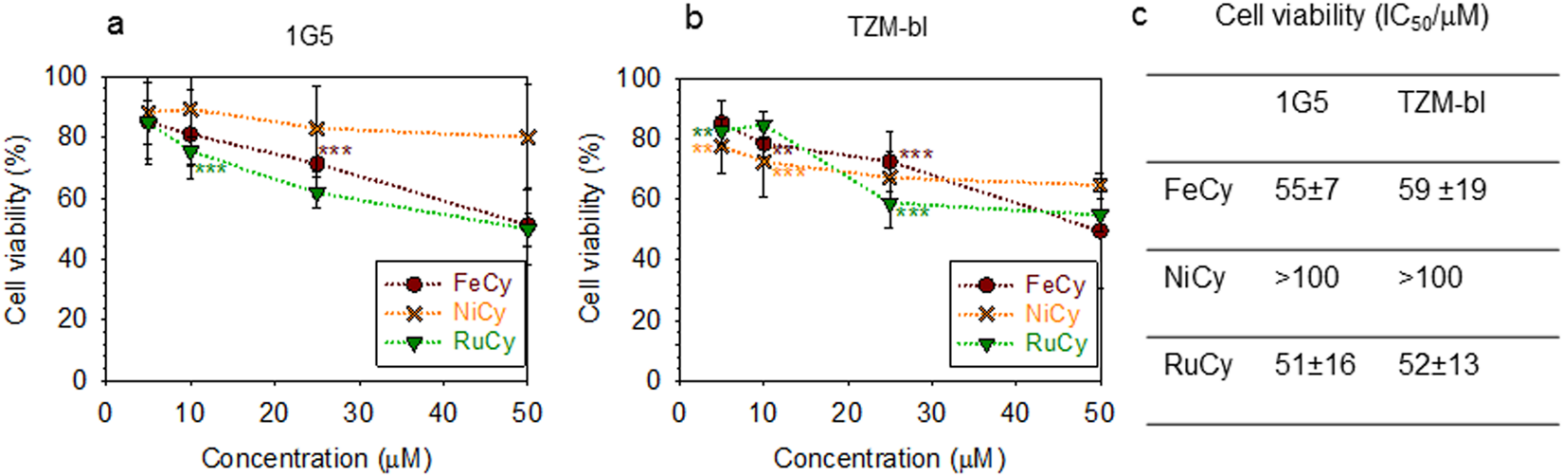
Cylinders have minimal effect on cell viability. 1G5 **(a)** or TZM-bl **(b)** cells were incubated with an increasing concentration of cylinders for 48 h and viability measured using an MTT assay. Data represent the mean ± s.d. of at least three independent experiments and data are plotted as percentage relative to control (cells in absence of cylinders, corresponding to 100% in each graph). Each y value was compared with the corresponding control by one way ANOVA analysis followed by Bonferroni post hoc test and P values < 0.05 indicate where their difference is statistically relevant. (*P < 0.05, **P < 0.01, ***P < 0.001). **(c)** IC_50_ values calculated by employing the complete cell viability curves in Figure S6.

To ensure that the anti-HIV activity is not mediated via the cylinders reducing cell viability we tested the cytostatic effect of FeCy, NiCy and RuCy cylinders for 1G5 and TZM-bl cells using an MTT assay (Fig. 5a,b and Figure S6). Although NiCy is not the most potent at inhibiting HIV-1 infectivity, it has least effect on cell viability. For RuCy cell survival rates of ~80% were noted at 5 and 10 µM concentrations at which RuCy has arrested viral replication. By contrast, FeCy does affect cell viability at the concentrations (above 25 µM) where it significantly affects anti-viral activity, rendering it the least appropriate of these three cylinder drugs.

**Figure 6:**
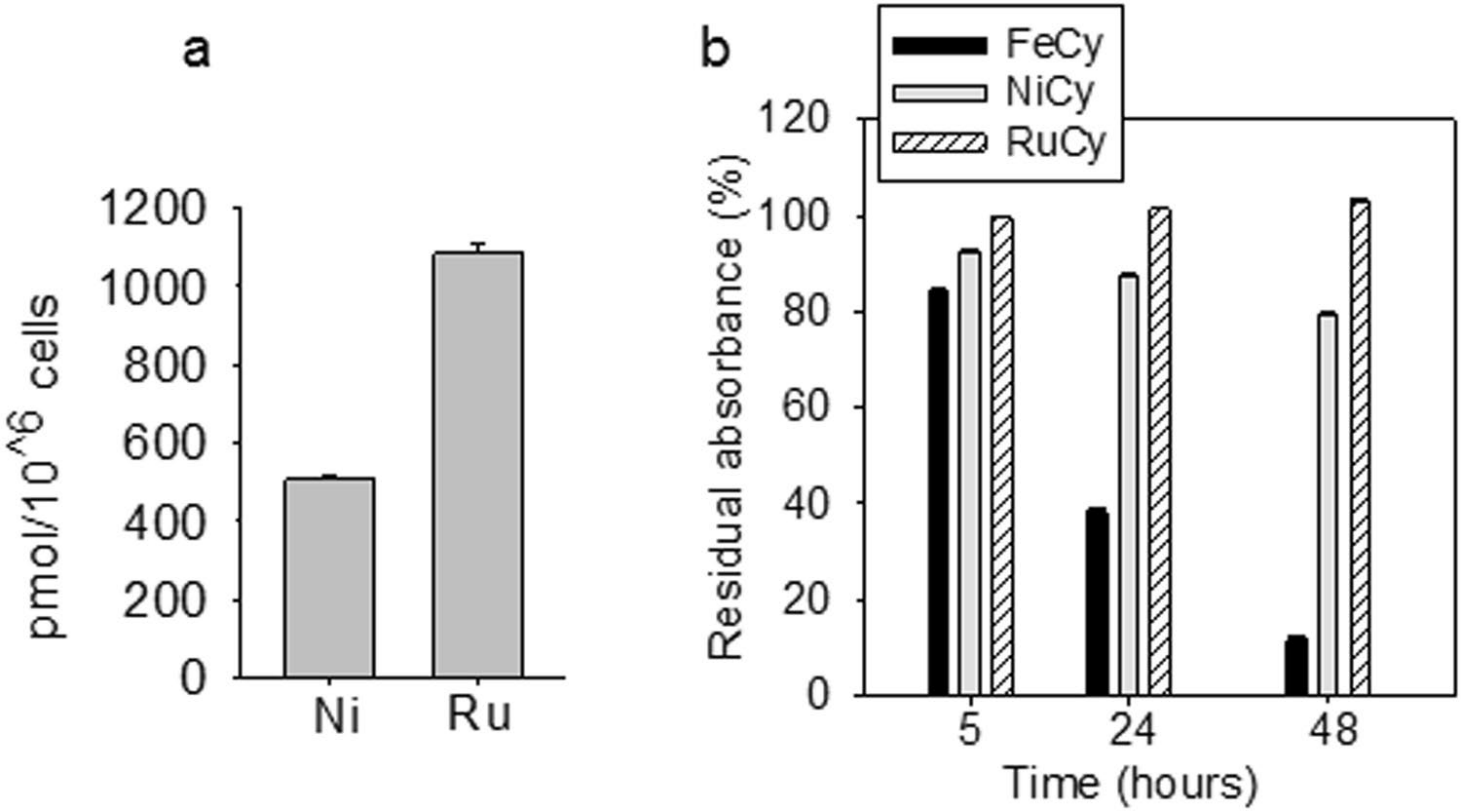
**(a)** Intracellular level of Ni and Ru. Concentrations of nickel and ruthenium detected in 1G5 cells by ICP-MS (pmol/10^6^ cells, data are mean ± s.e. of values from three independent experiments, *P* < 0.001). **(b)** Cylinder stability. % Residual absorbance of FeCy, NiCy and RuCy (at 574, 313 and 485 nm respectively) after 5, 24 and 48 hours on incubation at 37 °C in medium and in the presence of 10000 cells (data are mean ± s.d. of values from three independent experiments, see also Figure S7).

Since the three isostructural and isocationic cylinders show comparable TAR binding and inhibition of TAR-ADP-1 complexes (Figs 2 and 3) the differences in anti-viral activity may be explained by differential drug uptake/efflux or stability in cells. To investigate the level of cylinders within the cell, we measured the intracellular concentration of ruthenium and nickel by ICP-MS (inductively-coupled plasma mass spectrometry) in 1G5 cells after incubating with RuCy or NiCy (50 µM) for 16 h. Such analysis is imprecise for Fe-based drugs due to technical reasons and endogenous background. We observed 2-fold higher levels of intracellular ruthenium compared to nickel (Fig. 6a), that may contribute to the increased anti-viral activity of RuCy. To examine the stability of the metal cylinders we measured their UV-Vis absorption spectra in the presence or absence of cells over time (Figs 6b and S7). As would be expected from the known coordination chemistry of these metals, RuCy is stable under these conditions with no detectable change in absorbance over 48 h, whereas NiCy showed a 20% reduction in absorbance spectra over 48 h implying a half-life of approximately 1 week at 37 °C. In contrast, FeCy was more rapidly degraded with a half-life of less than 1 day. Whilst we know that cylinder stability is enhanced on binding nucleic acids^18,34^, it is noteworthy that the most stable cylinder showed the most effective anti-viral activity.

Both HIV-1 infection and cell viability assays suggest that RuCy is a candidate for further study. NiCy is also of interest since it shows lower anti-viral activity but with reduced effects on cell viability and thus the possibility of a different therapeutic window. In contrast, our results would not recommend further studies with FeCy as this compound showed negligible anti-viral activity at non-cytostatic concentrations.

Many viruses replicate their genomes using viral encoded enzymes that lack proof reading activity and generate viral diversity that enables escape from anti-viral drugs and host immune responses. Genetic polymorphism has been reported in the HIV-1 TAR region resulting in variable bulge base sequences and/or small variations in the number of bases in the bulge^35^. However, to maintain replicative fitness HIV requires the TAR RNA bulge motif for TAT recognition. Since the cylinder binds the bulge structure, rather than the sequence, and binds not only 3-base bulges but smaller and larger bulges^23^, it is unlikely that mutations in the TAR sequence will prevent cylinder binding. This new structural (shape-fit) nucleic acid targeting provides several advantages over more traditional approaches that target TAR RNA or TAT peptide. We have shown that such supramolecules inhibit HIV replication. Since the replication of many RNA and DNA viruses is dependent on secondary RNA structures^36^ we hypothesize that these cylinders will inhibit replication of a wide range of viruses. This opens intriguing new directions in the application of different supramolecular architectures to not only recognise different nucleic acid structure but to select their biological activity and address disease and infection.

## Methods

Synthesis purification and characterisation of Fe, Ni and Ru cylinders were carried out according to previously reported procedures^20,31,37^. Stock concentrations of compounds were prepared in sterile ultrapure water (from Sigma) for gel electrophoresis studies or in sterile PBS solution for cell biology experiments, by measuring absorbances with UV-Vis (Cary Varian 5000). TAR RNA sequence (5′-GGCCAGAUCUGAGCCUGGGAGCUCUCUGGCC-3′) was purchased from Integrated DNA Technologies (HPLC grade) and ADP-1 peptide (SFTTKALGISYGRKKRRQRRRPPQGSQTHQ-VSLSKQ) was purchased from Peptide Protein Research Ltd. (UK) with purity >98%. RNA and peptide concentrations of stock solutions were measured by UV absorbance employing the extinction coefficients provided by suppliers. All charts were prepared using SigmaPlot and statistical significance calculated using the ‘one way ANOVA’ analysis.

### Electrophoretic mobility shift assay

TAR RNA was radiolabelled at the 5′ end using T4 polynucleotide kinase and [γ-^32^P]ATP. RNA was annealed in 50 mM Tris HCl (pH 8.0) at 80 °C for 5 min and then chilled on ice for 10 min. For TAR-cylinder binding studies, RNA samples (2 µM) were incubated with increasing cylinder concentrations (1, 2, 3 and 4 µM) in TK buffer (Tris HCl (50 mM, pH 8.0), KCl (100 mM)) to a final volume of 10 µL for 30 min at RT and chilled on ice for 10 min. Samples were analysed by 15% non-denaturing polyacrylamide gel in 0.5x TB buffer (Tris HCl (40 mM), boric acid (45 mM)), pH 8.3 at 11 v/cm and 4 °C for 5 h.

For TAR-ADP-1 inhibition assays, 10 µL of TAR RNA (0.1 μM, radiolabelled and annealed as above) were pre-incubated with ADP-1 peptide (0.3 μM) in DTT (100 mM), 0.1% Triton X-100 and Tris.HCl (50 mM pH 8.0) for 15 min followed by incubation with cylinders (0.2, 0.4, 0.8 and 1.6 µM) for 30 min and chilled on ice for 10 min. Samples were run on a 10% non-denaturing polyacrylamide gel at 4°C for 2.5 h at 11 v/cm.

All mobility shift assays were performed employing the same starting radiolabelled TAR RNA solution and all gels were imaged by developing on a white phosphor screen and scanned using a Bio-Rad personal molecular imager (PMI) employing equal exposure conditions.

### RNA melting experiments

TAR RNA was dissolved in sodium phosphate buffer (10 mM, pH 7.0) containing EDTA (0.5 mM) and annealed by heating at 90 °C for 3 min followed by slow temperature decrease (30 min) down to 25 °C. 3 μM RNA were incubated with each cylinder (1:1 ratio) at 25 °C for 30 min (100 μL solutions). RNA melting experiments were performed by detecting absorbance at 260 nm when increasing the temperature from 25 to 95 °C, using masked microcuvettes (1 cm path length and 0.1 mL capacity) in a Cary Varian 5000 spectrophotometer equipped with a Peltier multicell block and temperature controller (1 nm bandwidth, heating rate 1 °C/min, 10 s average time). Melting temperatures (Tm, ± 0.5 °C) were calculated within the thermal heating program by applying a first derivative calculation and final Tm values are the average from 3 independent experiments.

### Fluorescent Intercalator Displacement

All measurements were carried in a Edinburgh Instruments FLSP920 fluorescence spectrometer (λ_ex_ 545 nm, λ_em_ 600 nm, dwell time 0.5 s) using a 3 mL quartz cuvette. The emission of TAR RNA (0.3 μM in Phosphate Buffer, pH 7.0, previously annealed as above) in the presence of Ethidium Bromide (EB, 1.2 μM) was measured and normalized to 0% fluorescence. Increasing concentrations of cylinders were added (from 0.1 to 2 equivalents in respect to TAR RNA) allowing 10 min stabilization before measuring emission. Each titration was repeated 3 times, reported as normalized variation of emission at 600 nm (ΔF) vs Cylinder/TAR ratio (Figure S2).

### Cell culture

1G5 cells were kindly provided by Professor Ariberto Fassati (University College London, London, UK) and cultured in RPMI medium supplemented with 10% FBS and 1% penicillin/streptomycin. TZM-bl cells were kindly provided by Professor Bill Paxton (University of Liverpool, Liverpool, UK) and cultured in DMEM medium supplemented with 10% FBS, 1% penicillin/streptomycin and 1% L-glutamine.

### HIV infectivity assay

For HIV-1 genesis and infection, 293 T cells were transfected with the pNL4.3 full-length HIV-1 plasmid using Fugene6 (Promega). Cells were transfected for 6 h at 37 °C and the media replaced with fresh DMEM supplemented with 3% FBS/1% penicillin/streptomycin. Subsequently, virus was collected at 48 h and 60 h post-transfection, the stocks were pooled, clarified by centrifugation at 2500 rpm for 10 min and stored at −80 °C. To evaluate the effect of FeCy, NiCy and RuCy on HIV-1 replication, 1G5 (100,000 cells in 100 μL) and TZM-bl (8′000 cells in 100 μL) were pre-treated with different concentration of cylinders (5, 10, 25, 50 μM) in 96 well plates, for 4 h prior to infection. Unbound virus was removed by washing and fresh media containing the cylinders was added. After 48 h cells were washed, lysed and luciferase activity measured.

### Cell viability assay

1G5 and TZM-bl (same cells/100 μL employed for infectivity assay) were treated with different concentration of cylinders (1, 2.5, 5, 10, 25, 50, 75, 140 μM in respective media) in 96 well plates, for 48 h at 37 °C. Cells were washed 3x with PBS and treated with MTT (3-(4,5-dimethylthiazolyl-2)-2, 5-diphenyltetrazolium bromide), 50 μg in 100 μL medium (final concentration) for 2 hours in incubator. The medium was removed, the purple crystals dissolved in 200 μL DMSO and absorbance at 576 nm was measured. IC_50_ values were calculated with SigmaPlot employing at least three repeats of triplicated measurements.

### Metal detection in cells by ICP-MS

1G5 cells (5 × 10^6^ cells in 5 mL medium) were incubated with Ru and Ni cylinder for 8 h at 37 °C (10 and 50 μM the 2 concentrations investigated). Cells were washed 3x with PBS by centrifugation, counted and cell pellets digested with nitric acid (~70%, Trace Select grade from Sigma-Aldrich) at 90 °C overnight. Solutions were diluted with water (Trace Select grade from Sigma-Aldrich) to achieve nitric acid concentration <5% and analysed by ICP-MS spectrometry (Agilent LC-ICP-MS (7500cx) at the University of Warwick, UK).

### Stability of cylinders by UV-Vis

50 μM of each cylinder were incubated at 37 °C for 48 hours in the presence of 10000 cells (either TZM-Bl or 1G5) or in medium only. Incubation was carried out in 96 well plates using 100 μL volume for each sample. UV-Vis profiles were monitored at 0, 5, 24 and 48 hours by using a CLARIOstar plate reader from BMG Labtech and the final results are the average of three independent experiments.

## Electronic supplementary material

Supplementary Information

## Data Availability

All data generated or analysed during this study are included in this published article (and its Supplementary Information files).
