# Supplementary material for: Metallo supramolecular cylinders inhibit HIV-1 TAR-TAT complex formation and viral replication in cellulo
Source: Sci Rep. 2018 Sep 6;8:13342. doi: 10.1038/s41598-018-31513-3 (PMC6127258; doi:10.1038/s41598-018-31513-3)
Supplement: Supplementary file 1 — Supplementary Information [file 41598_2018_31513_MOESM1_ESM.pdf]

Metallo supramolecular cylinders inhibit HIV-1 TAR-TAT complex formation and viral replication *in cellulo*

Lucia Cardo,[1†] Isabel Nawroth,[2†] Peter J. Cail,[1] Jane A. McKeating[3]* and Michael J. Hannon[1]*

[1] School of Chemistry, University of Birmingham, Edgbaston, Birmingham, B15 2TT, UK; [2] Institute of Immunology and Immunotherapy Centre for Human Virology, University of Birmingham, Edgbaston, Birmingham, B15 2TT, UK; [3] Nuffield Department of Medicine, Oxford University, Oxford, OX3 7BN, UK.

† Equal contribution from these authors.

*Correspondence to: m.j.hannon@bham.ac.uk; jane.mckeating@ndm.ox.ac.uk.

Supporting Information

Table of contents

Figure S1. **UV thermal assay** (page 2)

Figure S2. **Fluorescent Intercalator Displacement (FID) assay** (page 2)

Figure S3. **HIV TAR-ADP complex formation** (page 3)

Figure S4. **TAR-ADP complex inhibition** (page 3)

Figure S5. **Assessing the effect of cylinders on a luciferase-based assay** (page 4)

Figure S6. **Cell Viability assays** (page 5)

Figure S7. **Cylinder Stability** (page 6)

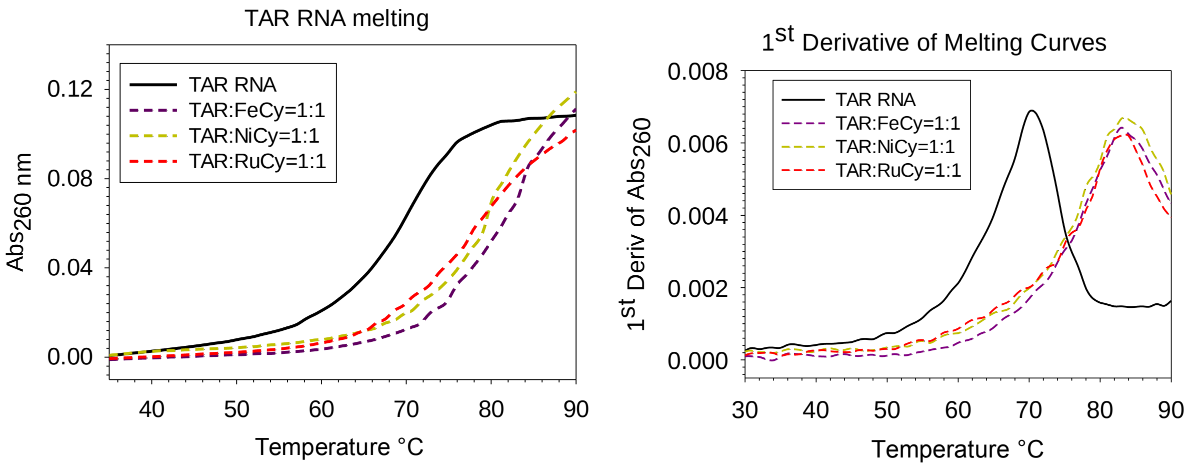

Figure S1. UV thermal assay. **(left)** Absorbance at 260 nm vs temperature increase of TAR-RNA (3 µM, in 10 mM Sodium Phosphate, pH 7.0, and 0.5 mM EDTA) alone (black solid curve) and in the presence of FeCy, NiCy and RuCy 1:1 ration (dashed curves). On the **right**, the corresponding 1st derivatives curves employed to calculate melting temperatures in Figure 2b. Each curve is the mean of three indipendent experiments.

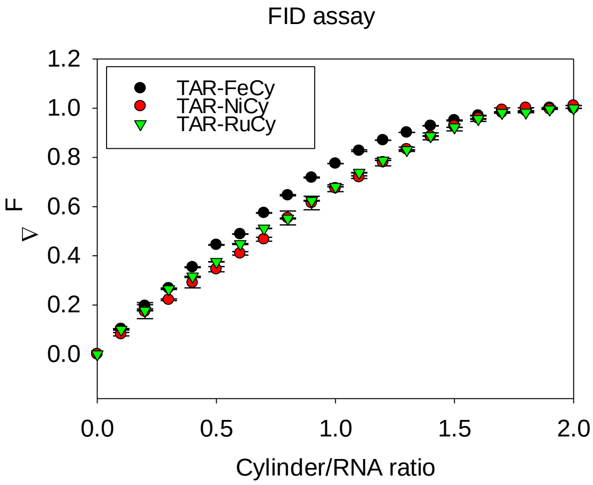

	**DC**$_{50}$ (µM)
FeCy-TAR	0.17±0.0
NiCy-TAR	0.21±0.1
RuCy-TAR	0.20±0.0

Figure S2. Fluorescent Intercalator Displacement (FID) assay. TAR-RNA (0.3 µM, in 10 mM Sodium Phosphate, pH 7.0) pre-treated with ethidium bromide (EB, 1.2 µM) was titrated with increasing concentrations of each cylinder (from 0.1 to 2 equivalents in respect of TAR-RNA). The chart shows variation of fluorescence in respect of emission of TAR-EB alone (normalized ΔF, λ_{ex} 545 nm, λ_{em} 600 nm) vs Cylinder/RNA ratio as a consequence of Ethidium Bromide being displaced from TAR RNA by cylinder. All three curves suggest that saturation is occurring after 1:1 ratio and data are the mean of three indipendent experiment ± s.d. **DC**$_{50}$ values (concentration of cylinder to riduce TAR-EB fluorescence by 50%) were calculated using the 'one site saturation' model by SigmaPlot.

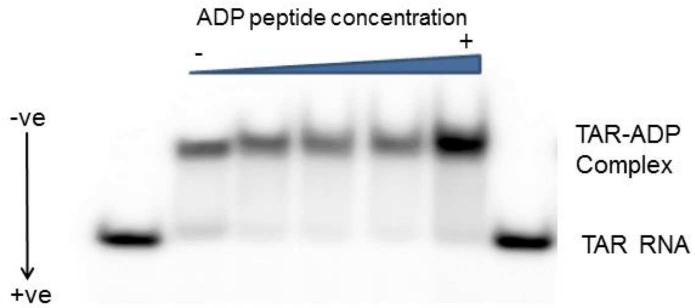

Figure S3. **HIV TAR-ADP complex formation**. Autoradiogram illustrating the adduct formed between TAR RNA (0.1 µM, first and last lanes) and the ADP-1 peptide. With increasing peptide concentration (0.2 – 1.0 µM), the intensity of the top band (corresponding to the adduct) increases.

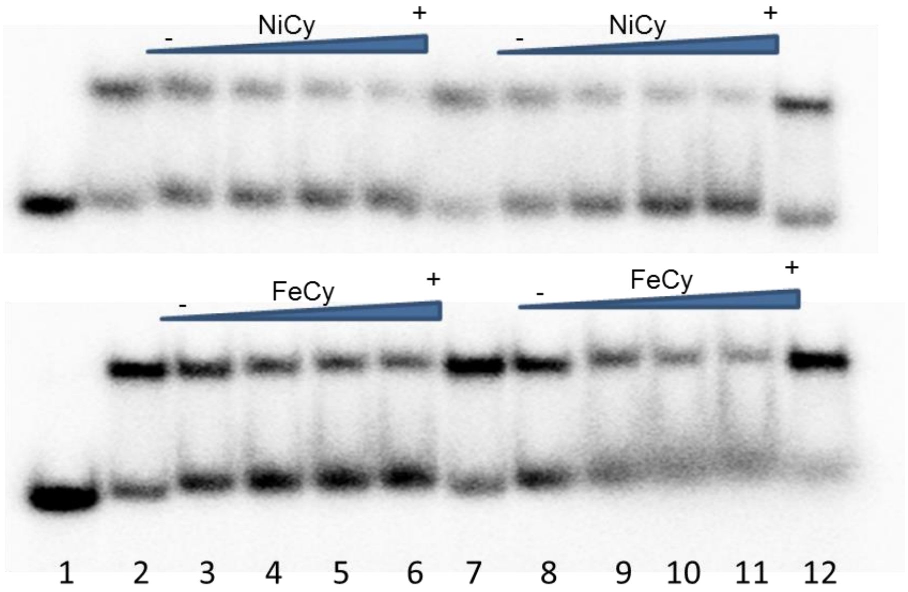

Figure S4. **TAR-ADP complex inhibition by NiCy (top) and FeCy (bottom)**. Full length version of the autoradiograms employed to generate figure 3.a Lanes 1-7 as described in the legend in Figure 3. Lanes 8-12 correspond to one repeat of the same experiment (using the same complex concentrations) on the same gel.

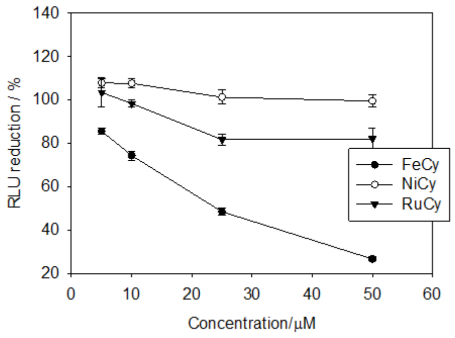

Figure S5. **Assessing the effect of cylinders on a luciferase-based assay**. The luciferin substrate reacts with the luciferase protein and emits light in the region of absorbance of cylinders (λ_{max} 574 and 485 nm for FeCy and RuCy respectively), therefore we assess whether the intensity of the light emitted during the assay is quenched or anyhow affected by the presence of cylinders. We employed TZM-bl cells because of their high basal LTR-luciferase expression in absence of HIV-1. TZM-bl (8000 cells) were treated with 50 µL of lysis buffer containing different concentrations of FeCy, NiCy and RuCy (5, 10, 25 and 50 µM, quadrupled for each concentration). 45 µL of lysate was treated with 45 µL of substrate and emission detected. The chart shows reduction of detected emission (%) vs concentration of complex in the lysis buffer. In this control the emission of luciferin is detected in large excess of cylinder compared to the measurement done during HIV infectivity assay (Figure 4), where cells are washed from excess of cylinder prior treatment with lysis buffer (only cylinders inside cells is present during the Infectivity assay). Considering this, we can confirm that NiCy and RuCy have no effect on the luciferase-based assay, whilst presence of FeCy causes reduction of luciferin emission only at highest concentrations (and when present in large excess).

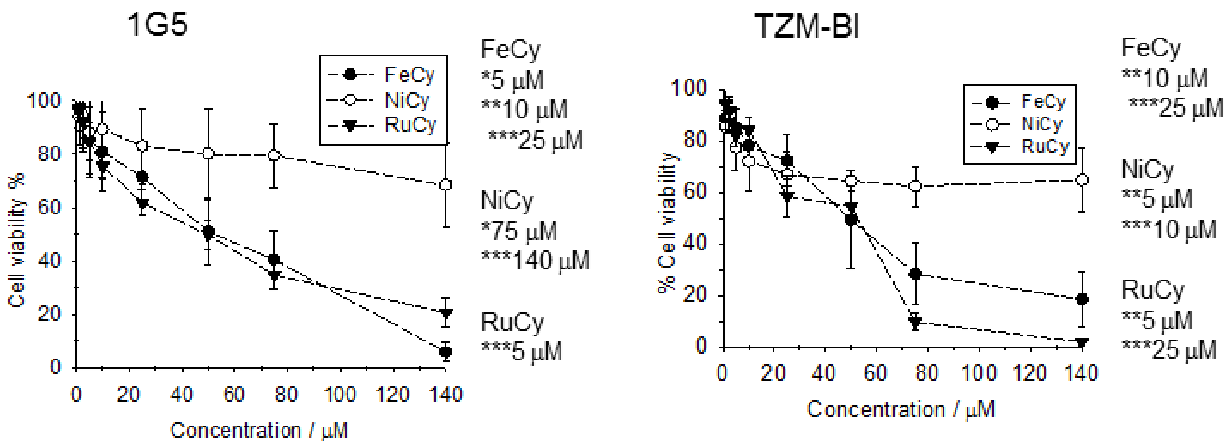

Cell survival (IC_{50}/µM)

	1G5	Tzm-bl
FeCy	55±7	59±19
NiCy	>100	>100
RuCy	30±10.0	51±13

Figure S6. Cell Viability assays. Complete cell survival curve by MTT assay in 1G5 (top left) and TZM-bl (top right). Cells were incubated with 8 different concentrations of cylinders (1, 2.5, 5, 10, 25, 50, 75 and 140 µM) for 48 hours. IC50s (table) and P values were calculated by processing data with SigmaPlot software. For each cylinder, all data are mean ±s.d. of at least three independent experiments (each containing triplicates for each concentration point); percentages are relative to cell viability of untreated cells (control). For each cylinder, P values indicate statistical differences between data point and control: $P > 0.05$= No symbol (no statistical differences), $P < 0.05$= *, $P < 0.01$= **, $P < 0.001$= ***

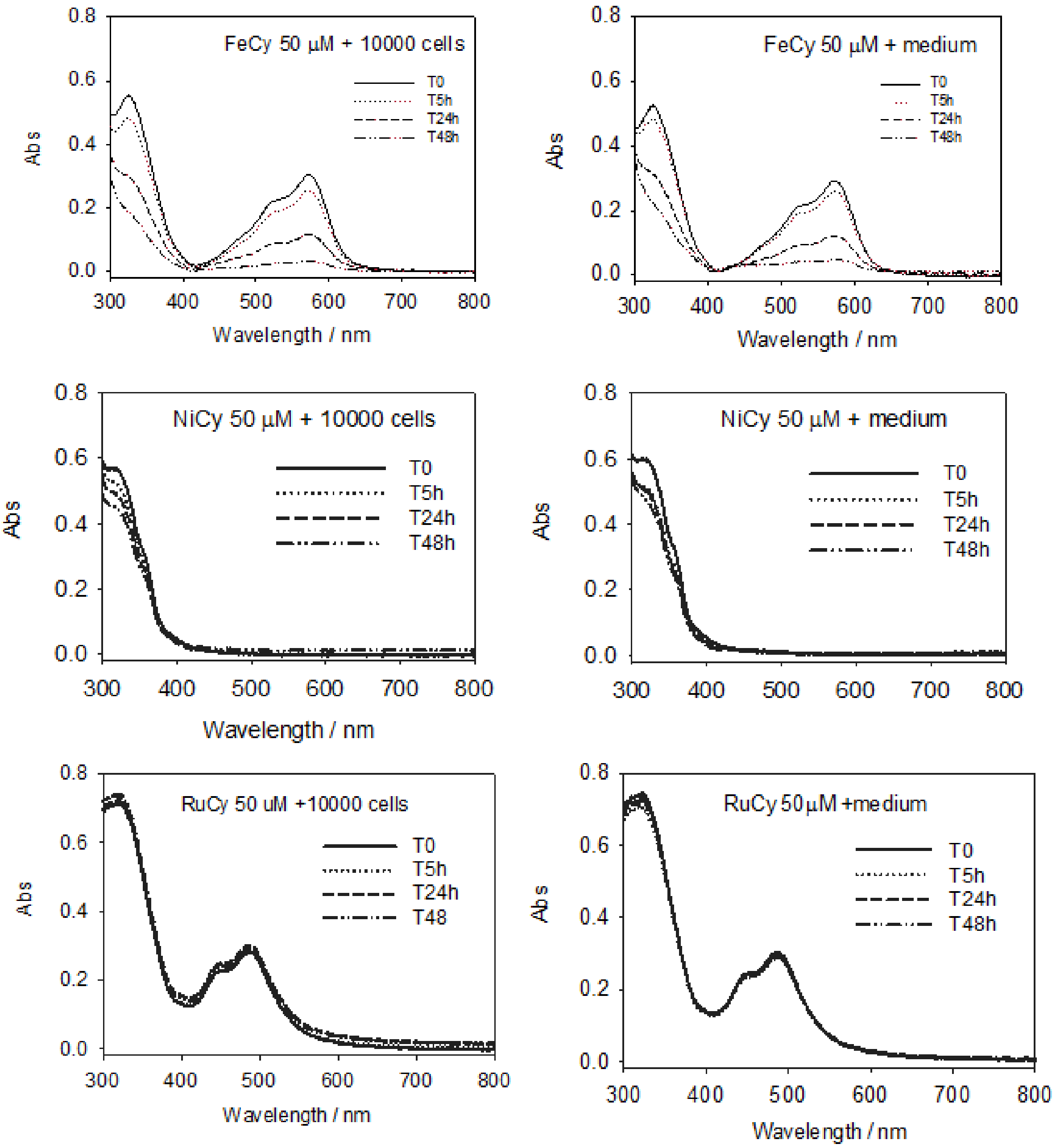

Figure S7. **Cylinder Stability.** To assess stability of FeCy, NiCy and RuCy in the conditions employed for biological assays, 50 µM of each cylinder were incubated at 37 °C for 48 hours in the presence of 10000 cells (either TZM-Bl or 1G5 produced the same results) or in medium only. Incubation was carried out in 96 well plates using 100 µL volume for each sample. UV-Vis profiles were monitored at 0, 5, 24 and 48 hours by using a CLARIOstar plate reader from BMG Labtech.
